# Late-Stage Diagnosis of Biliary Atresia in Rural America

**DOI:** 10.7759/cureus.101552

**Published:** 2026-01-14

**Authors:** Sofia Khan, Jacqueline Barsamian, Conner W Dunbar, Tetiana Litvinchuk

**Affiliations:** 1 Medical School, Alabama College of Osteopathic Medicine, Dothan, USA; 2 Pediatrics, Helen Keller Hospital, Sheffield, USA

**Keywords:** biliary atresia, bleeding varices, hepatomegaly, intraoperative cholangiogram, kasai hepatoportoenterostomy, pediatric liver transplant, rural, type iia biliary atresia

## Abstract

Biliary atresia (BA) is a rare neonatal disease characterized by progressive obstruction of the extrahepatic biliary system, with an etiology that remains poorly understood. This case describes the presentation of BA in a female neonate and underscores the consequences of delayed communication in a rural healthcare setting. The patient was born at a rural hospital, where laboratory tests were obtained but not processed and communicated to her pediatrician in a timely manner. As a result, her first notable clinical finding occurred at the two-week wellness visit, when mild hepatomegaly was suspected by physical exam, prompting an initial workup that was unrevealing. At subsequent visits, however, additional signs and symptoms emerged, leading to hospital admission for further evaluation due to concern for underlying liver pathology. The patient was diagnosed with type IIa BA with advanced cirrhosis, underwent liver transplantation, and at six months post-transplant, is doing well with a weight of 9.3 kg.

## Introduction

Between 1997 and 2012, biliary atresia (BA) was found in 4.47 per 100,000 infants in the United States, with a higher prevalence seen in females than in males [1]. This rare disease is characterized by progressive obstruction of the extrahepatic biliary system, resulting in jaundice, acholic stools, and, in advanced cases, a firm, enlarged liver. While etiology remains poorly understood, research has found that a direct bilirubin above a 1.0 mg/dL threshold has 100% sensitivity and good specificity (77%) for detecting BA in infants aged three to 60 days [2].

There are various types of BA, defined by the degree of obstruction present: type I BA occurs at the level of the common bile duct; type IIa BA occurs at the level of the common hepatic duct; type IIb BA affects the common bile duct, gallbladder, and common hepatic duct; and type III BA occurs throughout the biliary tree up to the porta hepatis [3]. The Japanese Biliary Atresia Registry (JBAR) uses a similar classification system with types I (atresia of the common bile duct), II (atresia of the common hepatic duct), and III (atresia at the porta hepatis, the most common type) [4]. The gold standard for diagnosis is an intraoperative cholangiogram, where contrast is injected into the gallbladder and visualized for obstructive flow via the intestine or biliary tree. If a biliary obstruction is visualized, the surgeon should perform a Kasai hepatoportoenterostomy (HPE) at that time [5]. In cases where patients present with BA and liver cirrhosis, Kasai HPE may not be performed due to the risk of fatal complications.

Furthermore, Kasai HPE performed within the first 30 days of life significantly reduces the chances of a near-future liver transplant, based on data from over 3000 cases in the JBAR [6]. This would require the detection of BA within the preclinical phase. While the etiology of BA remains unclear, links have been made to viral and genetic causes. BA cases with splenic involvement have higher incidences in the PRD1L1, CFC1, and FOXA2 genes [7-10]. Other notable causes include ingesting toxins, such as the isoflavonoid toxin isolated from the Dysphania plant, which was believed to have caused a BA epidemic in Australian goats [11].

In this case report, we present a female neonate diagnosed with BA following a progressive course of clinical findings and diagnostic evaluations. Initial laboratory studies obtained shortly after birth suggested hepatic dysfunction; however, the results were not processed or communicated promptly. This case highlights the challenges in rural U.S. healthcare systems, where limited access to subspecialists, delayed lab processing, and inadequate electronic communication can hinder timely interventions. Even with early detection, subspecialty referrals in rural areas may face weeks-long delays due to resource constraints [12]. This report outlines the patient's clinical course from birth to transplant referral and emphasizes the importance of early detection to improve outcomes.

## Case presentation

A 2.72 kg female neonate was born via C-section at 39 weeks of gestation due to breech position to a gravida 2, para 1 mother with no significant medical history. The delivery was uncomplicated, and the infant was fed adequately. Appearance, Pulse, Grimace, Activity, and Respiration (APGAR) scores were nine at both one and five minutes. The patient did not receive the hepatitis B vaccine and was discharged home at 2.69 kg. At her six-day newborn screening, she weighed 2.79 kg with no abnormalities, and the screening results were negative. Initial laboratory results showed a mildly elevated direct bilirubin level (1.2 mg/dL), with the total bilirubin level within normal limits; however, these results were not reviewed by the on-call physician or communicated to the patient's pediatrician until after subsequent symptoms had developed. At the two-week check-up, she weighed 2.99 kg; the physical exam revealed suspected mild hepatomegaly but no other abnormalities. An abdominal ultrasound showed no abnormal findings, including no triangular cord sign or atrophic gallbladder, which are typical indicators for BA. At the two-month well-child visit (day 63), the patient weighed 4.94 kg and presented with scleral icterus, jaundice, severe abdominal distention with rigidity, and caput medusae suggestive of portal hypertension. Due to concerns about a hepatic etiology, she was referred to the emergency room (ER) of a regional pediatric hospital.

In the ER, the patient was tachypneic and irritable. Abdominal ultrasound revealed free fluid but no mass or free air. She had acholic stools and vomiting alongside prior findings. Labs showed mildly elevated liver function tests (LFTs), procalcitonin, amylase, lipase, and direct bilirubin, with no leukocytosis. Intravenous (IV) antibiotics and dextrose 5% normal saline were started. Acute liver failure was ruled out, and the patient was transferred to the pediatric intensive care unit (PICU) with gastroenterology consultation.

**Figure 1:**
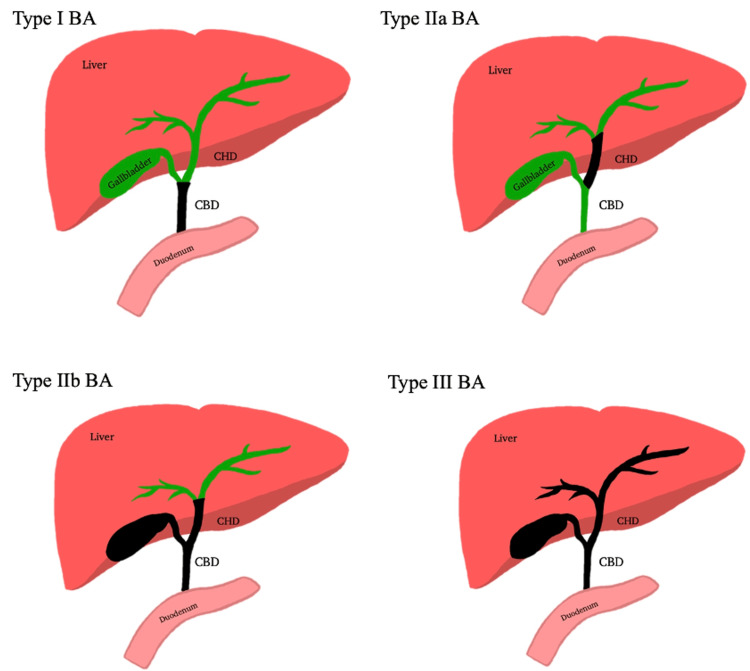
Types of biliary atresia Type I BA occurs at the level of the common bile duct; type IIa BA occurs at the level of the common hepatic duct; type IIb BA affects the common bile duct, gallbladder, and common hepatic duct; and type III BA occurs throughout the biliary tree up to the porta hepatis. BA: biliary atresia; CHD: common hepatic duct; CBD: common bile duct Original image made by Sofia Khan.

**Figure 2:**
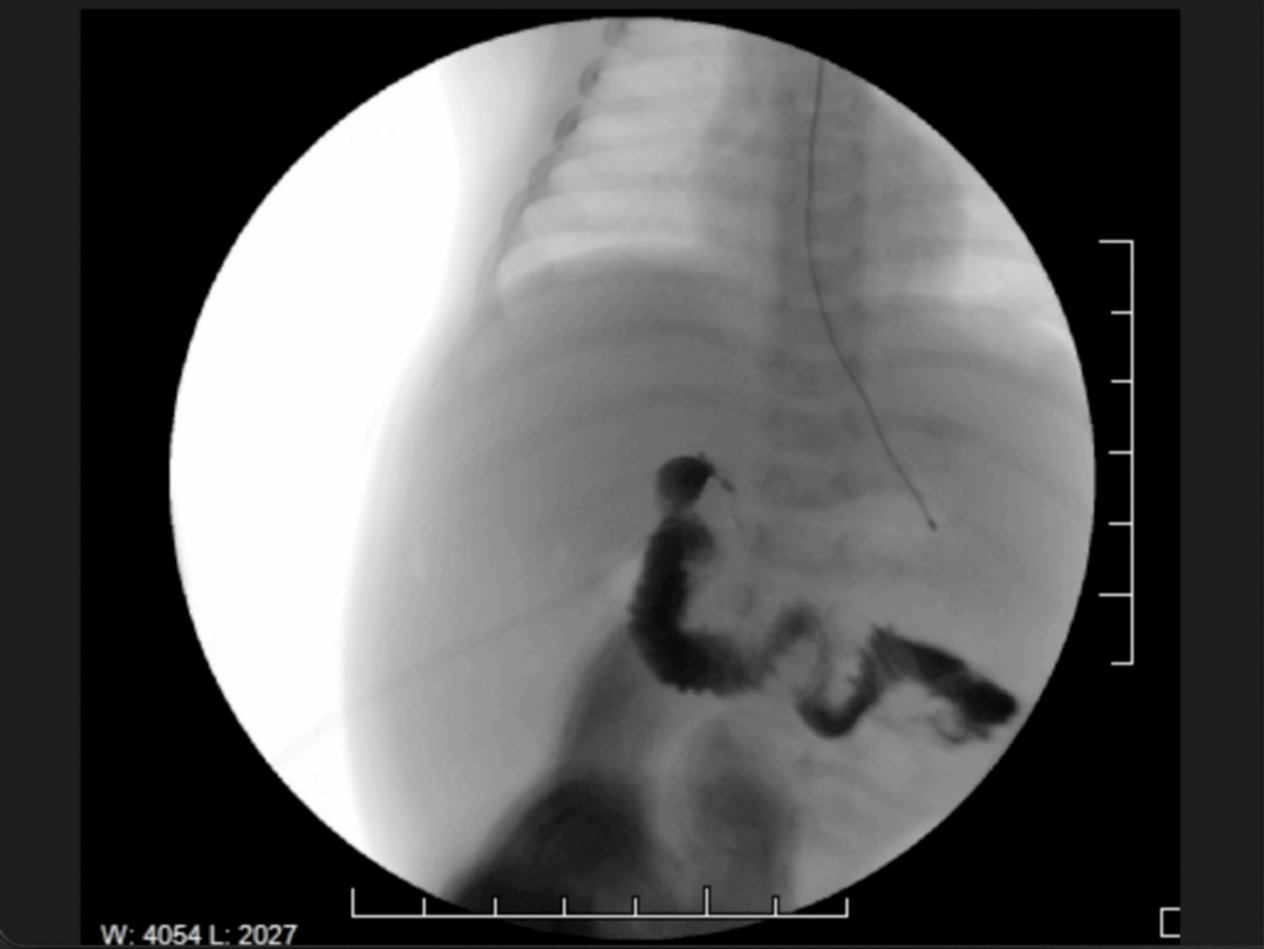
Type IIa biliary atresia confirmed with contrast injected into the biliary tree Contrast is injected into the biliary tree and fills distal structures (gallbladder, cystic duct, and common bile duct (CBD)), but does not pass through the common hepatic duct (CHD) into the liver or intrahepatic ducts. This confirms obstruction at the CHD level, consistent with type IIa biliary atresia, where the CHD is atretic while the distal ducts remain patent.

**Figure 3:**
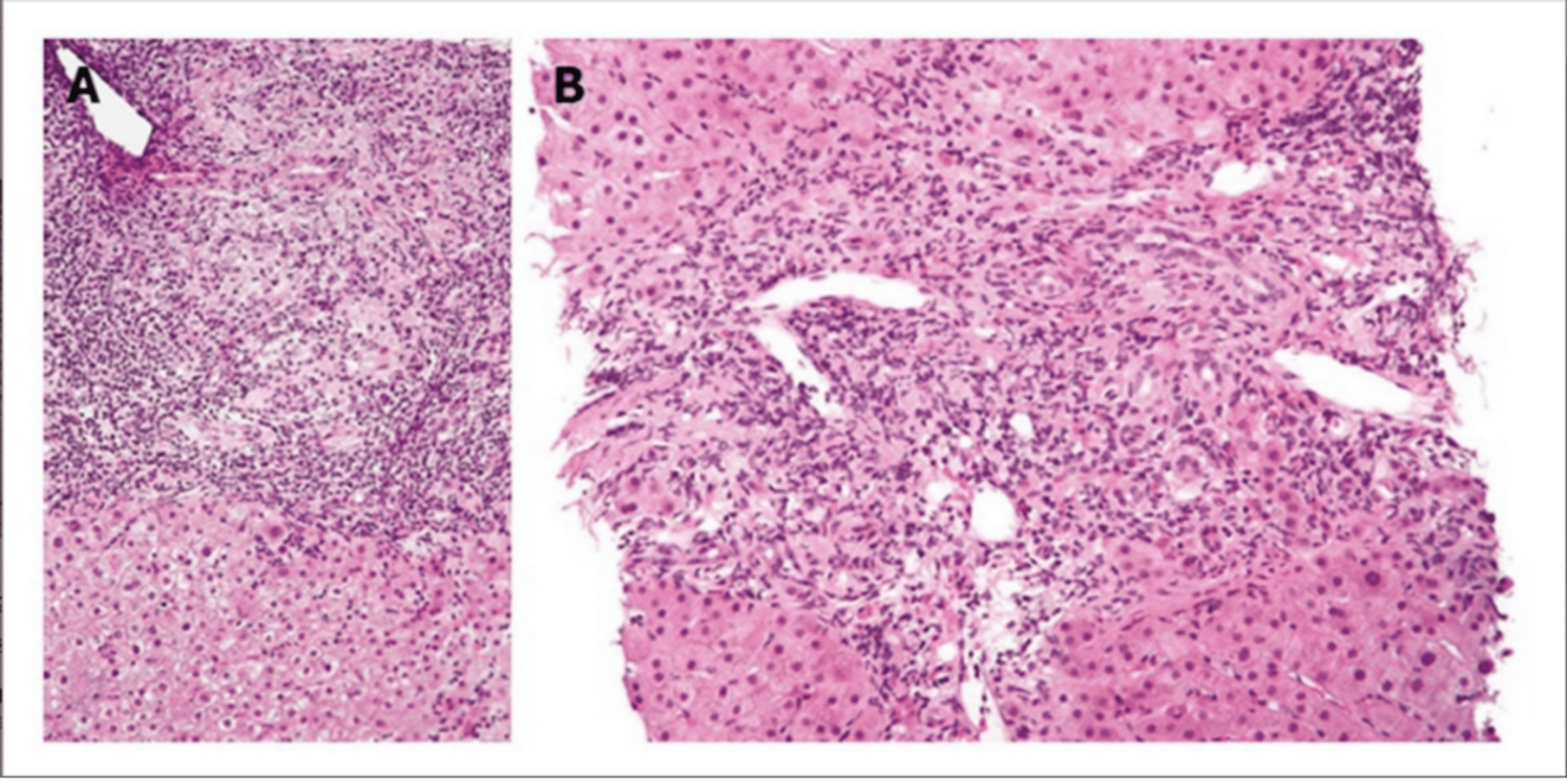
Photomicrographs of liver biopsy showing features of biliary cirrhosis Photomicrographs (A: x100 magnification; B: x400 magnification) showing features of biliary cirrhosis with a mixed inflammatory infiltrate (predominantly lymphocytes and plasma cells) concentrated around small intrahepatic bile ducts in the portal area, with relative sparing of hepatocytes (hematoxylin and eosin (H&E) stain). These features are representative of changes seen in progression from conditions such as biliary atresia. Reproduced from [13] under Creative Commons Attribution-Noncommercial 4.0 International License (CC BY-NC 4.0). No modifications were made.

Admitted to the gastroenterology service for 20 days (days 65-85), the patient received a neonatal peripherally inserted central catheter for total parenteral nutrition (TPN) with 1 mg vitamin K to address decompensated cirrhosis and malnutrition. She was also prescribed oral vitamin K (2.5 mg) and daily multivitamins. Liver ultrasound with Doppler showed a patent portal vein with sluggish hepatopetal flow but no anatomical abnormalities. High suspicion of BA prompted an intraoperative cholangiogram at approximately 70 days of age, large-volume paracentesis, and liver biopsy, revealing a cirrhotic liver and normal gallbladder. Contrast flow into the duodenum but not the hepatic duct confirmed type IIa BA (Figures 1, 2). Liver biopsy confirmed biliary cirrhosis with features of ductal plate malformation and severe fibrosis (Figure 3), and genetic testing (70-gene cholestasis panel) was negative [13]. Due to severe cirrhosis, Kasai HPE was deemed unsuitable. A percutaneous liver biopsy was not performed preoperatively, as it may not have definitively ruled out the need for exploratory laparotomy, given the high suspicion for BA and the gold standard diagnostic role of cholangiogram.

Post-procedure, the patient remained admitted for nutritional management with TPN and oral formula feeds. Ascites were managed with 25% albumin IV and dual diuretic therapy (furosemide and spironolactone) pending urgent liver transplant evaluation. The patient was placed on the transplant waitlist and discharged home, awaiting further management.

Eighteen days after discharge, the 103-day-old patient was readmitted on day 0 for central line placement to support adequate nutrition while on the liver transplant waitlist. Symptoms included worsening anemia, transaminitis, elevated gamma-glutamyl transferase (GGT), and prolonged international normalized ratio (INR). Hematochezia and anemia developed, prompting an esophagogastroduodenoscopy (EGD) revealing grade II esophageal varices. Hemoglobin (Hgb) decreased, requiring a packed red blood cell (pRBC) transfusion. Persistent hematochezia, suspected from rectal varices, was treated with octreotide infusion (1-4 mcg/kg/hr titration), IV Rocephin, and Protonix. The patient was transferred to the PICU, where bleeding resolved. Hepatic function tests remained elevated but stable, TPN progressed, and PO formula feeds advanced. Albumin and Lasix were administered for fluid balance. An EGD with sclerotherapy addressed multiple esophageal varices (grade I x4, II x2, III x1), with no rectal varices noted. Increased work of breathing and tachypnea prompted a viral respiratory panel (positive for rhino/enterovirus) and chest X-ray (moderate right pleural effusion due to hepatic hydrothorax), treated with albumin and Lasix. The patient was approved and transferred for a liver transplant.

The patient has been closely monitored by her surgical team and pediatrician. At six months post-liver transplant, she is doing well, with a reported weight of 9.3 kg. Her current medications include aspirin 81 mg chewable tablets once daily, cholecalciferol 25 mcg capsules daily, fludrocortisone 0.1 mg tablets daily, tacrolimus 1 mg/mL suspension (compounded) every 12 hours, and ursodiol 115 mg capsules twice daily. The patient's clinical course is summarized in Table 1. Laboratory results are detailed in Table 2.

**Table 1:** Timeline of the patient's biliary atresia disease course APGAR: Appearance, Pulse, Grimace, Activity, and Respiration; BA: biliary atresia; EGD: esophagogastroduodenoscopy; TPN: total parenteral nutrition; Hgb: hemoglobin; PICU: pediatric intensive care unit; PO: per os (by mouth/orally); CVL: central venous line; HA: hydrolyzed formula (in this context, "extensive HA 20 kcal/oz" refers to extensively hydrolyzed amino acid-based formula); CXR: chest X-ray; NPO: nil per os (nothing by mouth)

Time Point	Events and Interventions	Key Findings and Labs (H=High, L=Low, N=Normal; Pediatric Ranges)
Pregnancy	Unremarkable pregnancy.	-
Birth	C-section due to breech position. No complications.	Normal physical exam (PE).
Birth	Did not receive the Hep B vaccine.	-
Birth	APGAR: 9 (1 min), 9 (5 min).	-
Day 6	Newborn screening.	No abnormal findings.
Day 14 (2-Week Appointment)	2-week newborn appointment.	Palpable mild hepatomegaly (otherwise normal exam, no jaundice, no scleral icterus).
Day 14 (2-Week Appointment)	-	Abdominal ultrasound: no abnormalities.
Day 63 (2-Month Check)	2-month wellness checkup; sent to the emergency department.	Scleral icterus bilaterally, diffuse jaundice, abdominal distention with rigidity, and caput medusae.
Day 64	Admitted overnight.	Abdominal ascites.
Day 64	Started morphine 0.25 mL IV, Tylenol 15 mg/kg PR (once), ceftriaxone 75 mg/kg IV, and 5% dextrose normal saline IV.	-
Days 65-85	Transferred to PICU gastroenterology. Liver US with Doppler: patent portal vein, sluggish hepatopetal flow.	-
Days 65-85	Large-volume paracentesis. Liver biopsy: biliary cirrhosis.	-
Days 65-85	Started TPN, Vit K 1 mg IV, Vit K 2.5 mg/multivitamins PO daily, 25% albumin IV (x3), daily dual diuretics (spironolactone 9 mg BID, furosemide ~2.5-2.8 mg BID). Intraop cholangiogram: confirmed type IIa biliary atresia.	-
Days 65-85	-	Urgent liver transplant evaluation for type IIa BA with ascites/cirrhosis. Discharged home.
Day 107	Central line placement for nutrition support on transplant waitlist.	Worsening anemia.
Day 107	-	Hematochezia/anemia; EGD: Grade II esophageal varices.
Day 107	-	Hgb drop (L); pRBC transfusion.
Day 111	Transferred to GI service. Continued octreotide (1 mcg/kg/hr), Rocephin, IV Protonix. Stable condition.	-
Day 112	CVL incorrectly clamped, repaired bedside; stable for TPN. Hematochezia recurred.	-
Days 113-114	Hematochezia persisted; octreotide increased to 2 mcg/kg/hr + bolus. Ongoing bleeding.	-
Days 113-114	Formula feeds discontinued; Pedialyte by mouth.	-
Day 115	EGD with sclerotherapy (varices: Grade I x4, II x2, III x1 esophageal; no rectal).	Bleeding addressed; stable post-procedure.
Day 115	Hgb (L); 10 cc/kg pRBC (post: Hgb (N)). Lasix post-transfusion.	-
Day 115	Octreotide to 3 mcg/kg/hr; Rocephin x7 days.	-
Day 116	Bleeding ceased; octreotide reduced to 2 mcg/kg/hr. Pedialyte resumed. Stable hepatic function.	-
Day 116	Diuretics: Lasix 2 mg BID, spironolactone 5 mg BID (>200 cc + fluid).	-
Day 117	PO formula (extensive HA 20 kcal/oz) resumed; octreotide to 1.5 mcg/kg/hr. Vit K 5 mg IV; TPN Vit K increased. Stable hepatic labs.	-
Day 117	Increased breathing work/tachypnea (viral panel + rhino/enterovirus; CXR: mod R pleural effusion due to hepatic hydrothorax).	-
Day 117	Treated with albumin 1 g/kg + Lasix 0.5 mg/kg.	-
Days 117-118	Two bloody bowel movements (BMs); NPO. Octreotide to 3 mcg/kg/hr; diuretics increased (Lasix 2.5 mg BID, spironolactone 7.5 mg BID).	-
Days 117-118	Hgb (L); monitored for transfusion.	-
Days 118-119	Transferred to PICU (high bleeding risk).	-
Days 118-119	Rust-colored stool; octreotide to 4 mcg/kg/hr. Hgb stable; glucose (L); treated with D10% bolus (repeat (N)).	-
Day 119	-	Stable condition.
Day 119	Transferred for liver transplant.	-

**Table 2:** Laboratory tests with reference ranges Reference ranges are approximate for neonates/infants zero to three months, sourced from Mayo Clinic Laboratories pediatric reference values [14], University of Iowa Stead Family Children's Hospital Pediatric Reference Ranges [15], and general pediatric guidelines (including those informed by the American College of Clinical Pharmacy and similar sources for electrolytes, coagulation, etc.) [16]. GGT: gamma-glutamyl transferase; INR: international normalized ratio

Lab Test (Units)	Reference Range (Pediatric, 0-3 Months)	Birth	Day 63	Day 64	Day 85 (Discharge)	Day 107	Day 107 (Later)	Day 115	Day 115 (Post)	Day 116	Days 117-118	Days 118-119	Days 118-119 (Repeat)
Total Bilirubin (mg/dL)	1.0-12.0 (peaks ~3-5 days, then <5.0)	6.5	8.6	7.2	-	-	-	-	-	18.07	-	-	-
Direct Bilirubin (mg/dL)	0.0-0.6	1.2	6.2	1.2	-	-	-	-	-	15.22	-	-	-
Indirect Bilirubin (mg/dL)	0.6-10.5	5.4	-	-	-	-	-	-	-	-	-	-	-
Unconjugated Bilirubin (mg/dL)	0.6-10.5	-	-	6	-	-	-	-	-	-	-	-	-
Potassium (mEq/L)	3.7-5.9	-	5.0	-	5.8	-	-	-	-	-	-	-	-
Chloride (mEq/L)	98-113	-	108	-	112	-	-	-	-	-	-	-	-
CO₂ (mEq/L)	20-28	-	17	-	13	-	-	-	-	-	-	-	-
Blood Urea Nitrogen (BUN) (mg/dL)	4-19	-	10	-	18	-	-	-	-	-	-	-	-
Creatinine (mg/dL)	0.2-0.4	-	0.2	-	0.17	-	-	-	-	-	-	-	-
Glucose (mg/dL)	45-126	-	77	-	98	-	-	-	-	-	-	48	75
Calcium (mg/dL)	8.8-10.8	-	9.9	-	9.1	-	-	-	-	-	-	-	-
Anion Gap (mEq/L)	5-15	-	15	-	9	-	-	-	-	-	-	-	-
Calculated Osmolality (mOsm/kg)	275-295	-	277	-	-	-	-	-	-	-	-	-	-
BUN/Creatinine Ratio	10-20	-	50	-	-	-	-	-	-	-	-	-	-
Total Protein (g/dL)	4.6-7.4	-	4.5	-	-	-	-	-	-	-	-	-	-
Albumin (g/dL)	2.9-4.2	-	3.0	-	2.9	-	-	-	-	-	-	-	-
Alkaline Phosphatase (U/L)	82-383	-	1,064	-	-	-	-	-	-	8778	-	-	-
Aspartate Aminotransferase (AST) (U/L)	<50	-	259	-	-	247	-	-	-	182	-	-	-
Alanine Aminotransferase (ALT) (U/L)	<35	-	355	-	-	174	-	-	-	130.9	-	-	-
Procalcitonin (ng/mL)	<0.5	-	-	0.58	-	-	-	-	-	-	-	-	-
Ammonia (µmol/L)	21-95	-	-	45	-	-	-	-	-	-	-	-	-
Sodium (mmol/L)	134-144	-	-	-	134	-	-	-	-	-	-	-	-
Hemoglobin (Hgb) (g/dL)	9.0-14.0	-	-	-	-	7.4	6.9	7.3	8.5	-	7.5	-	-
GGT (U/L)	11-134	-	-	-	-	457	-	-	-	190	-	-	-
INR	0.9-1.6	-	-	-	-	1.4	-	-	-	1.6	-	-	-

## Discussion

This case highlights the challenges of diagnosing BA in a rural healthcare setting and the consequences of delayed communication and limited access to subspecialty care. In rural U.S. areas, hospitals often lack pediatric gastroenterologists on-site, leading to prolonged referral timelines. Even if an outpatient referral had been made early, appointments would have been scheduled weeks later. Additionally, delayed lab processing and inadequate electronic communication prevented a timely review of her abnormal direct bilirubin. The patient's initial neonatal labs demonstrated an elevated direct bilirubin, which exceeds the 1.0 mg/dL threshold identified in large cohort studies to have 100% sensitivity for BA in infants aged three to 60 days [2]. This abnormal result should have prompted an urgent referral to pediatric gastroenterology. Unfortunately, the lab was not reviewed by the on-call physician, nor was it communicated to the patient's pediatrician until after the patient developed clinical signs, including hepatomegaly, jaundice, and portal hypertension.

By the time of her two-month well-child visit, the patient presented with severe clinical manifestations, including scleral icterus, jaundice, abdominal distention with rigidity, and caput medusae. Subsequent evaluation at a regional pediatric hospital confirmed type IIa BA with biliary cirrhosis. Due to the degree of cirrhosis, Kasai HPE was not possible, necessitating urgent liver transplantation. During her hospital course, she experienced multiple complications, including ascites, malnutrition, variceal bleeding, and hepatic hydrothorax, which required intensive management with TPN, diuretics, albumin, octreotide, transfusions, and endoscopic interventions before transplant. There is ongoing controversy regarding primary liver transplantation versus the Kasai procedure in advanced cases, with some evidence suggesting primary transplant may offer better long-term outcomes in severe cirrhosis, though Kasai remains the first-line where feasible [17].

These barriers, including delayed communication, limited access to subspecialty care, and prolonged referral timelines, collectively contributed to a delayed diagnosis, with critical implications for prognosis, given that earlier intervention, such as the Kasai procedure before 60 days of age, is associated with improved transplant-free survival [6,18].

The need for urgent referral pathways for time-sensitive neonatal conditions is clear, as well as the need for improved communication and health information technology (IT) systems in rural hospitals to ensure that lab results are reviewed promptly and communicated to primary care providers [2,6]. Expanded access to subspecialty services or telehealth consultations should also be implemented to facilitate earlier evaluation and management when local resources are limited [19]. Addressing these barriers can help rural healthcare systems improve timely diagnosis and intervention for neonates with BA, potentially improving outcomes and reducing the morbidity associated with delayed treatment [11].

## Conclusions

In conclusion, this case underscores the critical need for a high index of clinical suspicion when evaluating neonates with subtle physical exam findings, such as mild hepatomegaly, as the consequences of missing BA can be devastating. The rapid progression of this patient's condition, from an unremarkable presentation at six days and 14 days to severe liver cirrhosis and portal hypertension by two months, illustrates the urgency of timely diagnosis and intervention. Given the grave outcomes associated with delayed recognition, including the need for urgent liver transplantation as seen in this case, it further emphasizes the importance of consistent follow-up and early referral to specialized care. Moreover, as BA remains the leading indication for pediatric liver transplants worldwide, this case underscores the ongoing need for enhanced newborn screening strategies and ongoing research into its etiology to improve early detection and management, ultimately enhancing outcomes for affected infants.
